# The role of ZnO in TeO_2_ thin films for optical and radiation dosimetry applications

**DOI:** 10.1016/j.heliyon.2025.e42664

**Published:** 2025-02-14

**Authors:** Idris M. Mustapha, Kolo T. Matthew, Olarinoye I. Oyeleke, Ibrahim Sharifat, Muhammad K. Abdul Karim, Suriati Paiman

**Affiliations:** a Department of Physics, Faculty of Physical Sciences, Federal University of Technology, P.M.B. 65 Bosso, Minna, Nigeria; b Department of Physics, Nasarawa State University, P.M.B. 1022, Keffi, Nigeria; c Department of Physics, Faculty of Science, Universiti Putra Malaysia, 43400 UPM Serdang, Selangor, Malaysia; d Functional Nanotechnology Devices Laboratory (FNDL), Institute of Nanoscience and Nanotechnology, Universiti Putra Malaysia, 43400 UPM Serdang, Selangor Malaysia

**Keywords:** Spray pyrolysis, Optical properties, I-V characteristics, Dosimetry response, Thin film, Tellurium dioxide, Zinc oxide

## Abstract

The role of doping a p-type TeO_2_ film with an n-type ZnO on its optical and X-ray dosimetric properties was investigated by experiments using (ZnO)_*x*_(TeO_2_)_1-*x*_ thin films (x = 0, 0.2, and 0.4 wt%). The physicochemical, optical, and electrical properties of (ZnO)_*x*_(TeO_2_)_*1-x*_ thin films grown on a soda-lime glass substrate by spray pyrolysis were studied. The XRD study revealed a polycrystalline structure of the films and weak diffraction peaks belonging to paratellurite TeO_2_ in all film samples. A peak shift was observed in (ZnO)_0.2_(TeO_2_)_0.8_ and (ZnO)_0.4_(TeO_2_)_0.6_, indicating the presence of ZnO in the TeO_2_ crystal lattice. The FESEM image revealed the grain size and roughness of the films, which decrease with increasing ZnO concentration. The film thickness determined by cross-sectional FESEM images are 14.00, 14.11, and 12.12 μm for TeO_2_, (ZnO)_0.2_(TeO_2_)_0.8_, (ZnO)_0.4_(TeO_2_)_0.6,_ respectively. The UV–Vis study revealed high transparency in the visible light regions and a slight decrease in band gap values as ZnO concentration increased. The FTIR study showed resonances corresponding to the symmetrical equatorial and asymmetrical axial stretching frequencies of the Te-O bonds and a stretching mode of a Zn-O bond. Raman spectra detected the existence of Raman-active modes of TeO_2_ for all films and ZnO in the ZnO-doped TeO_2_ thin film samples. The current-voltage characteristics technique, which investigates the behaviour of electrical properties, was used to measure the change in current as a function of X-ray radiation dose. The I-V characteristics showed increased induced current at 100 and 200 cGy/min dose rates for all film samples. Consequently, we confirmed that the physicochemical properties of TeO_2_ films could be improved by ZnO doping at a 40 % wt% concentration for maximum dose response and high transparency. These thin films have high sensitivity and can detect both low and high-energy radiation, making them useful in various radiation-related applications such as medical dosimetry, environmental monitoring, and radiation safety. TeO₂'s superior optical properties are enhanced by ZnO's wide bandgap high exciton binding energy, and radiation sensitivity making it a competitive candidate for the development of miniaturized thin-film dosimeter that can fit in portable gadgets such as smartphones.

## 1 Introduction

The utilization of ionizing radiation is widespread in scientific, medical, and industrial fields [[1], [2], [3]]. In addition, monitoring gamma irradiation is crucial for irradiation facilities and various other domains [3,4]. The discipline of dosimetry pertains to the quantification of doses. Dosimeters are employed when there is a noticeable, consistent, and measurable alteration in the material exposed to gamma rays. Various dosimeters are utilized for different objectives throughout the period, such as the extensively used dyed polymeric thin film dosimeters [2,5]. When subjected to irradiation, polymers undergo alterations in their physical and chemical characteristics, particularly in the case of films. These changes are attributed to the creation of induced cross-links, the detachment of chains, and the formation of fresh chemical bonds [2,6].

Materials at the nanoscale have drawn greater interest than materials in their bulk form in the modern scientific era because of their unique physicochemical, optical, and electrical properties [3,5]. The rapid development of optoelectronics has led to challenges in the search for novel nanoscale materials [2,4,5]. Potential new materials can be found through the co-doping of oxide semiconducting materials together and sometimes with ions, as reported in recent studies [1,[6], [7], [8], [9]]. Tellurium dioxide thin film, in both crystalline and amorphous forms, is considered to be an essential nanomaterial and has found applications in modulators [8], gas detectors [10], gamma ray sensors [11], solar cells [12], and other areas. The co-doping of TeO_2_ with metal oxides such as ZnO can increase carrier concentration and competitive conductivity for both TeO_2_ and ZnO in thin film form and has been investigated by researchers [13,14]. However, studies on the effect of doping on the physicochemical properties of TeO_2_ thin films have not been widely investigated [15,16].

TeO₂ is essential to this study because of its unique combination of high density, optical transparency, and chemical stability, making it ideal for photonics, advanced materials, and radiation detection applications. Its high atomic number improves interaction with radiation, increasing detection sensitivity and efficiency, and its optical and piezoelectric properties allow for superior performance in devices such as scintillators, waveguides, or lasers [10,13]. TeO₂ offers exceptional durability and functionality compared to alternative materials as a stand-alone material and a flexible host for doped systems. Its inclusion in this study is anticipated to drive significant advancements in material performance and enable novel applications in radiation science and optical technologies [8,15].

Zinc oxide has several applications in the semiconductor sector, including sensors [15], lasers [17], gamma sensors [18], and solar cells [19], due to its wide bandgap and large exciton binding energy (60 MeV) [20]. Furthermore, the control of thin film morphology through doping is also a significant issue, as the shape and size of nanoparticles can influence various properties of the prepared products [16,[21], [22], [23], [24], [25], [26]]. Doping ZnO with TeO_2_ can alter the properties of ZnO through oxygen defect passivation [22,27]. Few studies are available on the TeO_2_-doped ZnO film, but they are limited in their scope [8,13,14].

ZnO's incorporation into TeO₂ thin films creates a new material structure with improved radiation dosimetric and optical characteristics [13,14,28]. TeO₂'s superior optical properties, including its high refractive index and broad transparency, are enhanced by ZnO's wide bandgap, high exciton binding energy, and radiation sensitivity. Thin films with enhanced photoluminescence, adjustable optical bandgaps, and increased radiation sensitivity may be created attributable to this combination [13,24,29]. With the possibility of scalable production techniques, the resultant materials provide accurate radiation dosage measurement and a wide range of optical applications [12,19,30]. This study makes advanced technologies in nuclear safety, environmental monitoring, and medical imaging possible. The present work reports on a systematic analysis of the changes in the physicochemical, optical, and dosimetry properties of TeO_2_ thin films due to ZnO doping. Due to its versatility and cost-effectiveness, we adopted the spray pyrolysis method for thin film preparation. The X-ray effect probed the dosimetry response of the film on I-V characteristic measurements.

## 2 Material and methods

### 2.1 Synthesis

The precursor solution for zinc oxide was synthesized by dissolving 0.863 g of zinc acetate dehydrate (Zn(CH_3_COO)_2_.2H_2_O) in 58 ml of methanol (CH_3_OH). To stabilize the solution, 2 ml of acetylacetone (C_5_H_8_O_2_) was added, and the solution was stirred thoroughly for 15 min using a magnetic stirrer until it became transparent. Similarly, the precursor solution for tellurium dioxide (TeO_2_) was prepared by dissolving 0.638 g of tellurium dioxide powder in 40 ml of hydrochloric acid (HCl) with a 43 wt% concentration. The solution was stirred for about 15 min at 67 °C until it became dissolved and transparent using a magnetic stirrer hotplate. To prevent precipitation, 20 ml of methanol was added to the solution and stirred. Finally, (ZnO)_*x*_(TeO_2_)_1-x_ for *x* = 0, 0.2, and 0.4 wt% precursor solutions were prepared by simple mixing and stirring using a magnetic stirrer at room temperature. This technique eliminates precursor heat degradation and ensures consistent distribution of ZnO in the TeO₂ matrix, which is crucial for dependable thin-film characteristics [14]. According to Rao et al. [31], adding ZnO into TeO2 matrices improves material homogeneity by minimizing phase separation, typical in tellurite-based glasses. Jain et al. [32] discovered that ZnO may improve radiation sensitivity via forming defects and electronic trapping processes and acting as a modifier. The motivation for adjusting ZnO concentrations is to achieve a synergistic balance supported by theoretical frameworks in the literature and actual data. The solution was kept at room temperature for about 24–48 h before the thin film deposition process to attain stability, homogeneity, and clear precursor solutions. The substrates used were soda-lime glasses, which were first washed in detergent, rinsed with distilled water, cleaned in ethanol, and finally rinsed with distilled water and dried before the deposition process.

### 2.2 Deposition

The (ZnO)_*x*_(TeO_2_)_1-*x*_ thin film deposition for *x* = 0, 0.2, and 0.4 wt% precursor solutions were carried out using the spray pyrolysis technique. An automatic ultrasonic spray pyrolysis coating equipment (U-spray USP 1500) was used with a software program (Repetier Host: Version 2.1.6, installed on a computer) to spray the solution onto the substrates. The airflow rate, solution flow rate, and nozzle-to-substrate distance were set at 0.2 kg cm^−2^, 0.15 ml min^−1^, and 3.0 cm, respectively. The heater-to-substrate temperature was kept constant at 300 °C. 1.2 ml of precursor solution was sprayed onto 10 × 10 mm^2^ of the substrate during each deposition process. After deposition, the thin film was removed and cooled to room temperature.

### 2.3 Characterisations

The structural information of the (ZnO)_x_(TeO_2_)_1-x_ thin films was studied using a Philips X-ray diffractometer (Model: PW 3040/60 MPD X'Pert High Pro Panalytical) with Cu-*K*α radiation (*λ* = 0.15 nm) at a scan step of 0.05° (2θ), data point counting time of 10.16s, and operated at 30 mA and 40 kV for the 2θ value of 20° to 80°. The optical properties of (ZnO)_*x*_(TeO_2_)_1-*x*_ were studied by measuring and analyzing the transmission optical spectra for photon wavelengths ranging from 200 nm to 900 nm at room temperature using a Shimadzu (Model: UV-3600) UV–Vis spectrophotometer. The film surface morphology was investigated by FEI (Model: Nova Nanosem 230) field emission-scanning electron microscopy (FESEM) at 50,000× magnification. The cross-sectional FESEM image of the (ZnO)_*x*_(TeO_2_)_1-*x*_ thin film samples was determined to measure the film thickness. An energy-dispersive spectrum (EDX) analysis of the films was performed using the FESEM. Both images of the film surface and cross-sectional area of the film were produced for morphological studies and film thickness determination, respectively. The functional groups were determined by a Bruker (Model: Alpha II) Fourier-transform infrared spectrometer, in which the IR spectra recorded were in the wavelength range of 4000 to 400 cm^−1^. The vibrational properties of the films were investigated by Raman high-resolution spectra collected at room temperature using a WITec (Model: Alpha 300 R) instrument with an Ar + laser line (*λ* = 488 nm) as the excitation source recorded in the region of 50–1200 cm^−1^ at the backscattering geometry. The current-voltage characteristics technique investigates the behaviour of the electrical properties by measuring the change in the induced current as a function of X-ray radiation dose. The dose is the energy absorbed by a material or tissue per unit mass, and the voltage is the electrical potential difference applied to the circuit. The X-ray radiation-induced changes in the electrical properties of (ZnO)_*x*_(TeO_2_)_1-*x*_ thin films were studied by measuring the I-V characteristics induced current of the films during irradiation at 100 cGy min^−1^ and 200 cGy min^−1^ dose rates using an Elekta synergy platform (Model: 153059) 6 MV photon linear accelerator.

## 3 Results and discussion

**Fig. 1:**
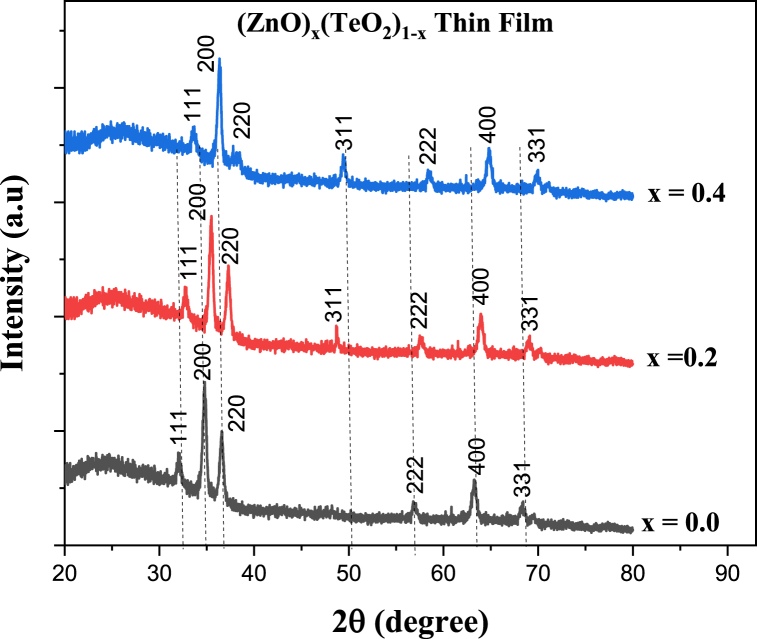
XRD pattern of (ZnO)_*x*_(TeO_2_)_1-*x*_ thin films.

The (ZnO)_*x*_(TeO_2_)_1-*x*_ thin films (*x* = 0, 0.2, and 0.4 wt%) structural properties were studied by the XRD spectra shown in Fig. 1. The spectra show a polycrystalline structure in all the samples. Peaks of diffraction related to the substrate material (soda lime glass) were observed in the XRD pattern. Peaks in the XRD patterns are located at 32.67°, 35.59°, 37.25°, 48.75°, 57.59°, 64.08°, and 69.09°, corresponding to the crystallographic planes (111), (200), (220), (311), (222), (400), and (331), respectively. The interplane spacings and diffraction patterns match the standard diffraction pattern of paratellurite TeO_2_ centred at an average peak position of 32.67° in all the thin film samples and the wurtzite ZnO thin film structure centred at an average peak position of 48.75° in TeO_2_-doped ZnO thin film samples ((ZnO)_0.2_(TeO_2_)_0.8_ and (ZnO)_0.4_(TeO_2_)_0.6_). Both orientations are consistent with the hexagonal wurtzite structure of JCPDS card No. 36–1451 for ZnO and the paratellurite structure of JCPDS card No. 75–2087 for TeO₂ [25,26]. The results support ZnO and TeO₂'s distinctive diffraction patterns. The increase in concentration of ZnO in the thin film samples has led to slight changes in peak positions (shift) when compared with the pure TeO_2_ film sample. It can be seen that the diffraction peaks were intense and narrower, implying the good polycrystalline nature of the (ZnO)_*x*_(TeO_2_)_1-*x*_ thin films and that the thin film samples have a high purity of paratellurite phase TeO_2_ and wurtzite phase ZnO.

Doping-induced lattice distortion could be responsible for the peak shift in the XRD spectra of TeO₂ and TeO₂-doped ZnO thin films. This is because Te⁴⁺ ions have a higher ionic radius than Zn^2^⁺, which results in compressive or tensile strain. Grain size or texture changes, defect development (such as oxygen vacancies or interstitials), tension and strain from the substrate and the film's mismatched thermal expansion could all contribute to the shift.

To calculate the inter-planar spacing (*d*-spacing), the Bragg's law relation was used as shown in Equation (1) [18,25];

(1) 2dsinθ=nλ

where λ=1.54Å is the wavelength of $CuK_{\alpha }$, θ= diffraction angle and *n* = diffraction order. The relation by Debye-Scherrer was used for the mean crystallite size calculation shown in Equation (2) [16,[25], [26], [27]];

(2) $$D=\frac{k^{\prime }\lambda }{\beta \;\cos\;\theta }$$

where β is the radian FWHM, $k^{\prime }$ ≈ 0.9 is the shape factor, and λ is the *CuKα* wavelength radiation, respectively. The Gaussian function fits the XRD patterns to determine FWHM peaks. The radian FWHM values of TeO_2_ thin films decreased in (ZnO)_0.2_(TeO_2_)_0.6_ and increased in (ZnO)_0.4_(TeO_2_)_0.6_ with increasing ZnO concentration.

Table 1 displays the average full width at half maximum and the average crystallite size as calculated. The augmentation of ZnO concentration in the TeO_2_ film samples resulted in an enhancement of crystallinity and a reduction in crystallite size. The decline in estimated crystallite size with increased ZnO concentration may be attributed to the merging of crystallites in the thin films and the greater atomic weight and ionic radii of Te^2+^ than Zn^2+^ [14].

**Table 1:** Some structural parameters of (ZnO)_*x*_(TeO_2_)_1-*x*_ thin films.

Mean peak position (^o^)	Lattice plane (h k l)	Mean *d*-spacing (nm)	Mean FWHM (^o^)	Mean crystallite size (Å)
32.67	(1 1 1)	31.6	0.197	421
35.59	(2 0 0)	27.4	0.354	236
37.25	(2 2 0)	19.3	0.118	710
48.75	(3 1 1)	13.7	0.393	222
57.59	(2 2 2)	15.8	0.315	288
64.08	(4 0 0)	13.7	0.394	238
69.09	(3 3 1)	12.5	0.630	153

**Fig. 2:**
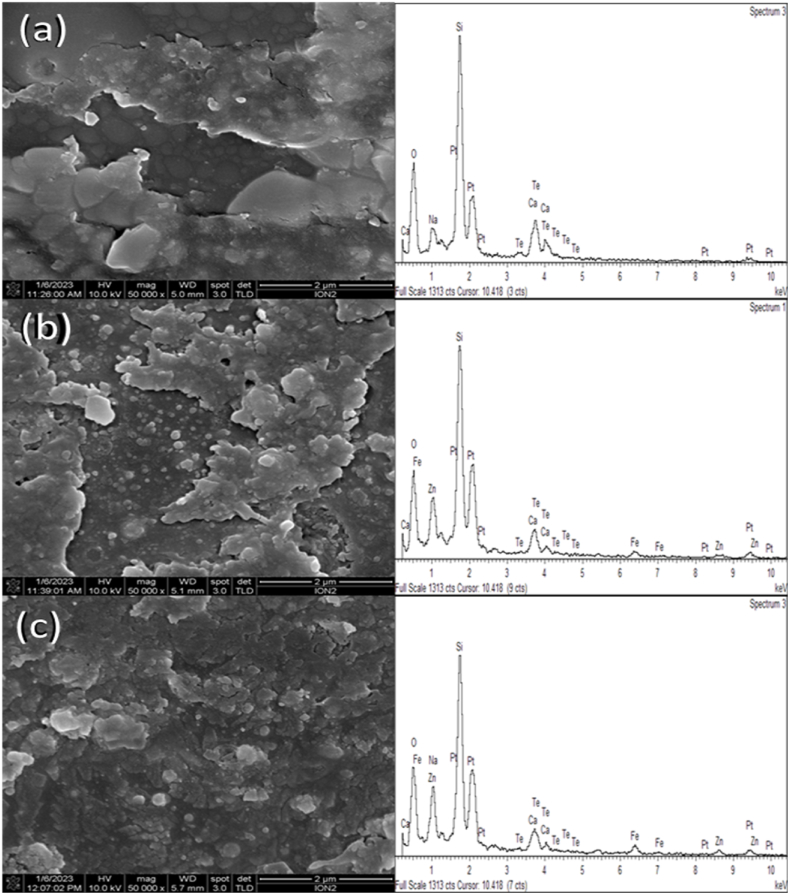
FESEM image with EDX spectra of (a) TeO_2_ thin film (b) (ZnO)_0.2_(TeO_2_)_0.8_ thin film (c) (ZnO)_0.4_(TeO_2_)_0.6_ thin film.

The FESEM images of (ZnO)_*x*_(TeO_2_)_1-*x*_ for *x* = 0.0, 0.2, and 0.4 wt% are presented in Fig. 2, which were used to examine the morphology of the films at 50,000× magnification. The images reveal a distribution of non-uniform clustered structures, with the stacked TeO_2_-doped ZnO thin layers together. As a result, agglomerated nanoparticles within the film are seen separately, with non-uniform shapes and non-crystalline grains. However, as the amount of ZnO doping increases, the particle sizes decrease, resulting in a more uniform distribution of the films' nanoparticles. Thus, the morphology of the TeO_2_ film is improved by ZnO doping. In addition to the surface morphology, the film thickness determined by cross-sectional FESEM images are 14.00, 14.11, and 12.12 μm for TeO_2_, (ZnO)_0.2_(TeO_2_)_0.8_, (ZnO)_0.4_(TeO_2_)_0.6_ respectively. In comparison to pure TeO₂, the modest increase in thickness for (ZnO)_0.2_(TeO_2_)_0.8_ may improve its optical absorption and radiation sensitivity while preserving a comparable level of transparency. The decreased thickness in (ZnO)_0.4_(TeO_2_)_0.6_, however, may be explained by variations in surface energy or solution viscosity during the deposition process [28]. Although there may be a slight decrease in radiation absorption, the optical clarity and mechanical stability of this thinner layer are increased. These differences emphasize how crucial it is to balance thickness-related effects for certain optical and dosimetric applications by adjusting ZnO content.

In order to create a specimen map, EDX is used in conjunction with FESEM to examine the kinds and qualities of elements present on the surface of the film nanomaterial (Fig. 2). Given that X-rays originate in a 2-μm deep region, the electron beam is moved across the sample during an EDX examination to create an image of the elements present. Usually, this procedure takes many hours. Since the film is positioned on the surface of the substrate, the EDX then measure the composition and amount of heavy metal ions present in the film. However, elements with an atomic number lower than 11 are difficult for EDX to detect. The TeO_2_-doped film spectrum confirmed the presence of the peaks corresponding to Te, O, and Zn in the Zinc-doped film. The quantitative weight ratios of the elemental compositions in the investigated films are given in Table 2. The weight ratios calculated are close to the nominal stoichiometry within the error range experiment. Furthermore, the spectrum shows that the intensity of the ZnO peak increases with increasing ZnO doping, indicating the film's stoichiometry and that Zn ions have been effectively in the lattice of the TeO_2_ semiconductor. The elements such as Si, Na, Ca, and Pt are derived from the substrate and buffer coating. The Zn/O and Te/O_2_ ratio was less than 1 compared to the perfect chemical stoichiometry of the ZnO and TeO_2_. These indicate the presence of imperfections (oxygen vacancies) in the ZnO-TeO_2_ thin film. A similar observation has been reported by Silambarasan et al. [23].

**Table 2:** Elemental concentration in mol% of energy dispersive X-ray spectrometry analysis.

Sample	Film element (mol%)			Substrate element (mol%)			Buffer layer coating (mol%)
	Te	Z	O	Si	Ca	Na	Pt
T:0Z	16.53	0.00	32.31	23.6	4.06	3.20	20.30
T:20Z	8.09	5.66	28.54	21.49	4.12	4.09	28.01
T:40Z	7.21	9.04	26.99	22.05	3.79	3.40	27.52

**Fig. 3:**
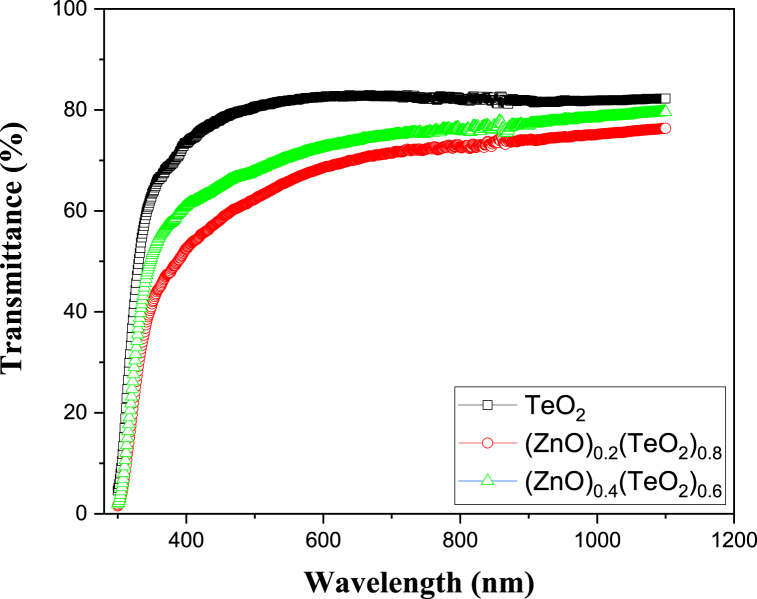
Plot of UV–Vis spectra of (ZnO)_*x*_(TeO_2_)_1-*x*_ thin films.

The optical properties of (ZnO)_*x*_(TeO_2_)_1-*x*_ thin film for *x* = 0, 0.2 and 0.4 wt% were examined using UV–Vis spectra shown in Fig. 3. ZnO decreases the transparency in TeO_2_-doped ZnO thin film samples. Since the variations in transmittance also depend on the effect of doping, the reduction in transparency is related to the film's structural properties, which are dependent on the material characteristics of the film. The undoped TeO_2_ thin film sample has the highest transparency among all the thin film samples investigated [15,17]. This trend is related to the low surface roughness and high carrier concentration. Factors affecting the absorption coefficients of TeO_2_ thin films include (1) the number of incoming photons entering the thin film is influenced by the surface roughness; (2) dopant content usually causes a change in lattice arrangement symmetries, which alters the propagation of wave and photons and increases scattering (the degree of lattice distortion caused by the dopant content of the thin films affects the optical path of rays inside the thin films); (3) the number of photons absorbed might vary depending on the carrier concentration in the medium. The unit volume of film has a high carrier density and could also result in a high probability and number of absorbed photons. In summary, thin films with high absorption coefficients exhibit characteristics of low roughness, a low degree of diffuse reflection, high incident photons, and a high probability of photons being absorbed.

The transmittance spectrum between the wavelengths of 700–900 nm shows slight fluctuations due to the interference of reflected light between the film-substrate and air-film interfaces. This indicates that the film has low surface roughness and good uniformity. The average transparency of the films are 79 %, 62 %, and 68 % for TeO_2_, (ZnO)_0.2_(TeO_2_)_0.8_, and (ZnO)_0.4_(TeO_2_)_0.6_, respectively. The lower transmittance observed in (ZnO)_0.2_(TeO_2_)_0.8_ and (ZnO)_0.4_(TeO_2_)_0.6_ thin films indicates increased photon absorption, which can increase the material's sensitivity to radiation by enhancing greater interaction between incoming radiation and the film. Higher transmittance observed in pure TeO₂ film ensures better optical clarity, which is advantageous for applications requiring minimal signal attenuation, such as optical dosimetry.

The transmittance results in this study are higher than 25 % [24] for undoped ZnO micro rods and 60 % [27] for undoped TeO_2_ micro sausages, and lower than 80 % [6] for ZnO-Te thin film. A sharp absorption edge was observed at approximately 300 nm, which agreed with a study by de Godoy et al. [32]. Furthermore, there is a significant relationship between the band gaps and the absorptions during which incoming photons will gather enough energy to cause the excitation of electrons across the band gaps [29,33]. The transmittance measurements can obtain the absorption coefficient of the (ZnO)_*x*_(TeO_2_)_1-*x*_ thin films. Swanepoel's method was used to calculate the coefficient of absorption of the film since it is void in the absorption strong zone, using equation (3) [6]:

(3) $$\alpha =\frac{1}{t}\ln\left(T\right)$$

where α, *T* and *t* are coefficients of absorption, spectra of transmittance and thin film thickness, respectively. Close to the absorption edge (in the absorption strong zone) of the transmittance, there is a relationship between α and the optical energy gap (*E*_*g*_), which follows from the power-law behaviour of Tauc's relation [15]:

(4) $$\left(\alpha h\nu \right)=A{\left(h\nu -E_{g}\right)}^{r}$$

where *h* ν is the photon energy, *A* is a constant, and the exponent *r* = $\frac{1}{2}$ indicates that direct transitions are allowed since the linear graph is seen near the band edge, which is the best.

**Fig. 4:**
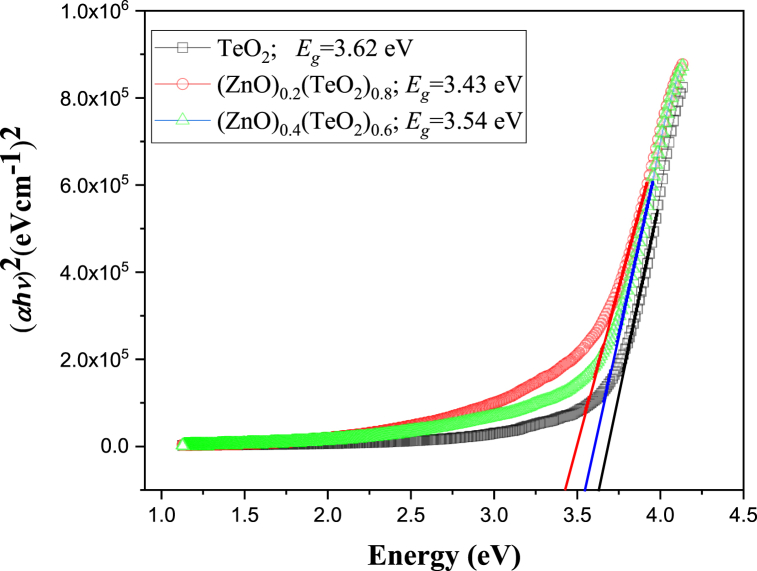
Plot of ${\left(\alpha hv\right)}^{2}$ versus energy of (ZnO)_x_(TeO_2_)_1-x_ thin films.

The estimated optical band gap (*E*_*g*_) of the films is 3.62, 3.43, and 3.54 eV for TeO_2_, (ZnO)_0.2_(TeO_2_)_0.8_, and (ZnO)_0.4_(TeO_2_)_0.6_, respectively, determined using the Tauc plot of the ${\left(\alpha h\nu \right)}^{2}$ versus Energy (*hν*) shown in Fig. 4. The optical band gap of the films decreases with an increase in ZnO concentration. Burstein can explain the broader optical band gap energy of the thin films–Moss effect, which is attributed to the energy of transition in degenerate semiconductors due to the partially filled conduction band [15,32]. The oxygen vacancy (V_O_^0^) generates a defect-localized state (DLS) well inside the band gap, which localizes two electrons, according to Lany and Zunger's model [29]. In a setup with a Zn-Zn distance of 4.0, the Zn neighbours relax outward when the two electrons at the DLS of V_O_^0^ are optically stimulated to V_O_^2+^. The DLS of V_O_^2+^ becomes empty due to the DLS being moved towards the conduction band's interior. Additionally, the conduction band above the conduction band minimum (CBM) is where the DLS expands and resonates. A perturbed host state (PHS) and a metastable conductive state (MCS) would form at the CBM due to the DLS states' resonance with the conduction band.

**Fig. 5:**
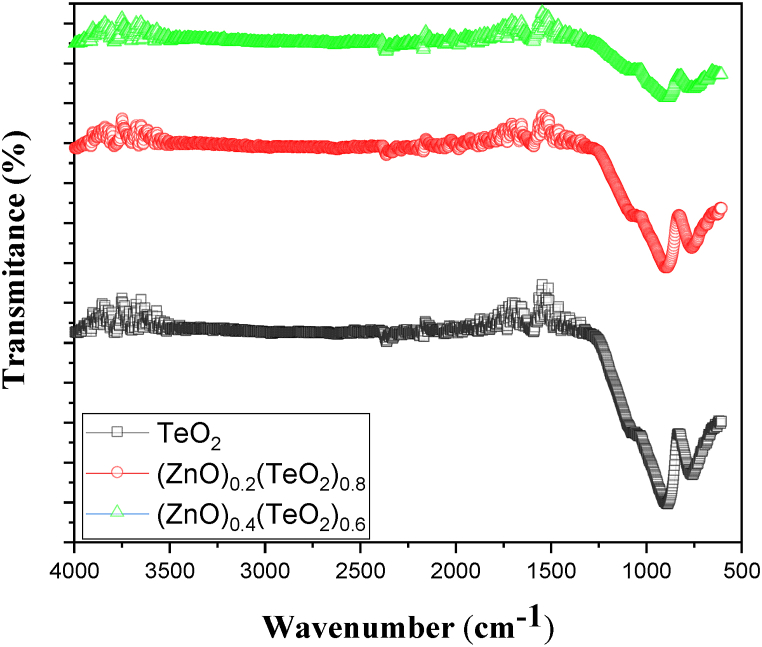
Plot of FTIR spectra of (ZnO)_*x*_(TeO_2_)_1-*x*_ thin films.

Fig. 5 displays the typical FTIR spectra of (ZnO)_*x*_(TeO_2_)_1-*x*_ thin films for x = 0, 0.2, and 0.4 mol% recorded in the 4000-500 cm^−1^ range for chemical stretching and bonding analysis. The FTIR spectrum of TeO_2_ thin film includes the typical absorption bands of TeO_2_, centred at wavenumbers of 880 and 745 cm^−1^, which is comparable to the equatorial symmetrical and asymmetrical axial frequencies stretching of the Te–O bonds, respectively, according to Refs. [34,35]. It can be observed that the two peaks of absorption centred at wavenumbers 880 and 745 cm^−1^ decrease with an increase in ZnO concentration. The bands in the wave number region of 600–1200 cm^−1^ are due to Zn-O bonding [9]. The growth of a small absorption peak at 675 cm^−1^ in both (ZnO)_0.2_(TeO_2_)_0.8_ and (ZnO)_0.4_(TeO_2_)_0.6_ is attributed to the stretching mode of a Zn-O bond. The peak at 670 cm^−1^ corresponds to the Te-Zn-O bond formation [36]. Furthermore, a small absorption band due to the OH vibrational stretching mode, centred at 3663 cm^−1^ wavenumber, was detected, indicating partial hydration of the oxide phase. CO_2_ stretching was seen at 2359 cm^−1^ for all the thin film samples [36,38], which could be attributed to the absorption of CO_2_ during characterization. The FTIR bands around 1700 cm^−1^ correspond to C=O vibrations, as reported by Gawai et al. [37]. The FTIR study confirms that ZnO was successfully doped into the TeO_2_ site.

**Fig. 6:**
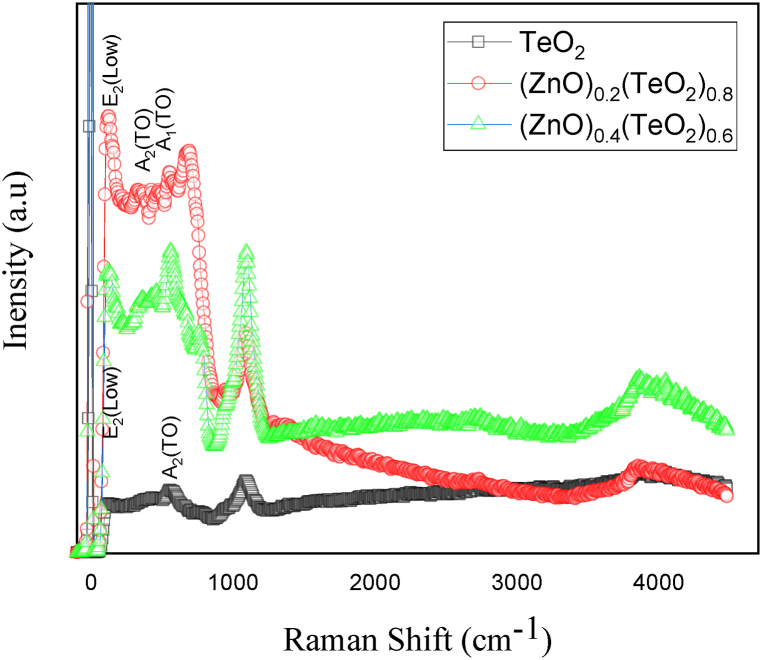
Micro Raman spectra of (ZnO)_x_(TeO_2_)_1-x_ thin films.

Fig. 6 displays the typical Raman spectra of (ZnO)_*x*_(TeO_2_)_1-*x*_ thin film for *x* = 0, 0.2, and 0.4 wt%. The TeO_2_ primitive cell consists of 12 atoms, and the 33 lattice vibrations longwave are shared between the species of symmetry as follows: ${9A}_{1}+{8A}_{2}+{8B}_{1}+{8B}_{2}$. They all correspond to Raman's active mode. The corresponding eight modes of vibrations of the eight shortest (equatorial) Te–O bonds are in the expected interval of 500–850 cm^−1^, which is the stretching modes region of the TeO_2_ lattices [[40], [41], [42]]. ZnO with wurtzite structure belongs to the $C_{6v}^{4}\left(p6_{3}mc\right)$ space group, one of the simplest uniaxial crystals. For the perfect crystal of ZnO, only the optical phonons at Γ point of the Brillouin zone are involved in first-order Raman scattering. Group theory predicts the existence of the following optic modes: $\Gamma_{opt}{=A}_{1}+E_{1}+{2E}_{2}+{2B}_{1}$ [42,43]. The polar modes *A*_1_ and *E*_1_ are split out into longitudinal optical (LO) and transverse (TO) phonons, where both modes are infrared and Raman active. *E*_2_ modes are non-polar and are only Raman active. *B*_1_ modes are Raman and infrared silent.

Raman spectra of (ZnO)_*x*_(TeO_2_)_1-*x*_ samples (*x* = 0, 0.2, and 0.4 wt%) were investigated, and the ranges are similar based on the second derivative of the relative spectrum search for hidden peaks. The TeO_2_ thin film (TeO_2_) peak at 558 cm^−1^ is assigned to the A_2_ (TO) mode [38,39]. Similarly, these peaks were found in all of the spectra studied here. The peaks at 132 cm^−1^ in the (ZnO)_0.2_(TeO_2_)_0.8_ film and 120 cm^−1^ in the (ZnO)_0.4_(TeO_2_)_0.6_ film are assigned to the E_2_ (low) mode corresponding to ZnO [41,42]. The slight differences in peak positions are associated with intrinsic defects such as oxygen vacancy, zinc interstitial, and other defects [39,40]. Also, the apparent peaks at 688 cm^−1^ in the (ZnO)_0.2_(TeO_2_)_0.8_ film and 564 cm^−1^ in the (ZnO)_*x*_(TeO_2_)_1-*x*_ film are attributed to the A_1_ (TO) mode corresponding to TeO_2_. On the other hand, the peak around 580 cm^−1^ (688 cm^−1^ in (ZnO)_0.2_(TeO_2_)_0.4_ film and 564 cm^−1^ in (ZnO)_0.4_(TeO_2_)_0.6_ film) could be assigned to the E_1_ (LO) mode of ZnO [42]. However, some authors have observed this peak in forbidden scattering geometry in samples doped with N, associated with resonantly enhanced LO phonons due to the breaking of Zn-O bonds [38,41,42]. The peak values detected around 99 cm^−1^ to 130 cm^−1^ in E_2_ (low) mode indicate the characteristic wurtzite structure of ZnO in (ZnO)_0.2_(TeO_2_)_0.8_ and (ZnO)_0.4_(TeO_2_)_0.6_ films. Similar observations have been reported by Akan et al. [38]. Overall, the Raman analysis detected the existence of Raman-active modes of TeO_2_ for all the films and ZnO in the (ZnO)_0.2_(TeO_2_)_0.8_ and (ZnO)_0.4_(TeO_2_)_0.6_ thin film samples [38,39,42].

**Fig. 7:**
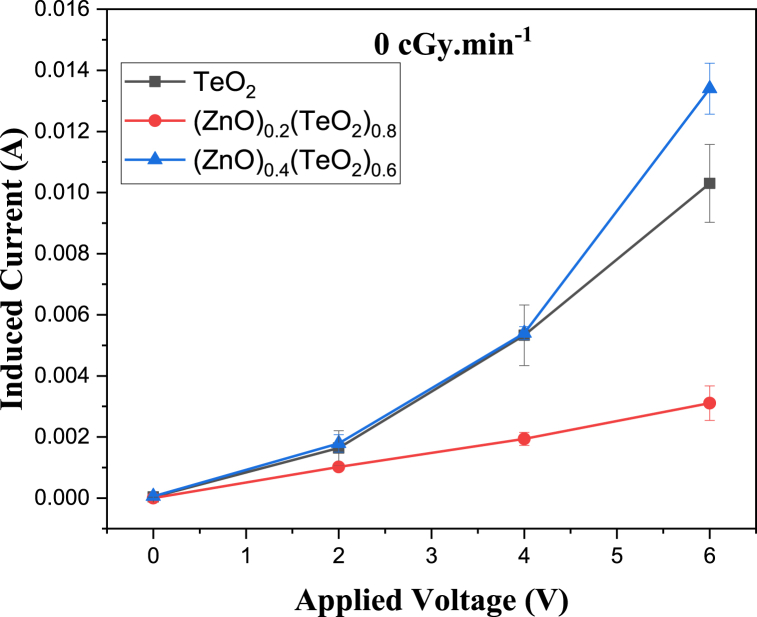
I-V characteristics plot of (ZnO)_x_(TeO_2_)_1-x_ thin film at 0 cGy min^−1^ X-ray dose rate.

**Fig. 8:**
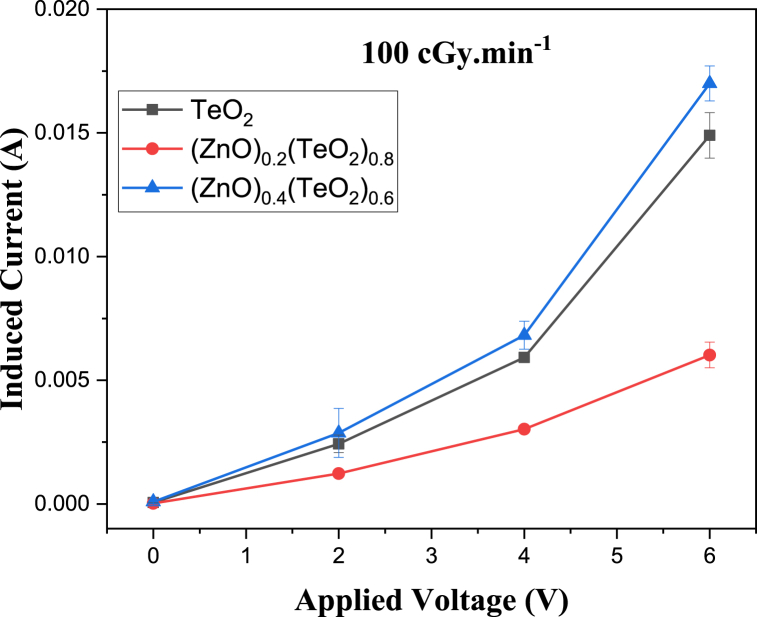
I-V characteristics plot of (ZnO)_x_(TeO_2_)_1-x_ thin film at 100 cGy min^−1^ X-ray dose rate.

**Fig. 9:**
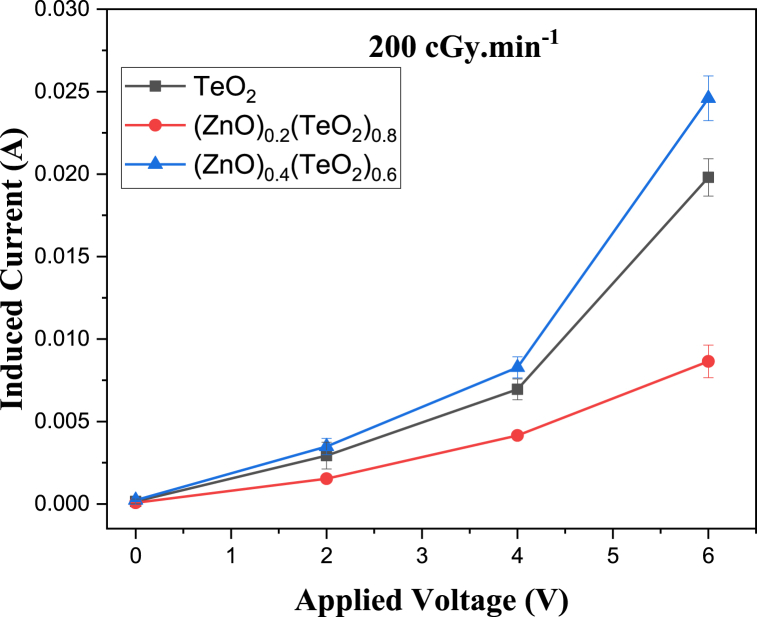
I-V characteristics plot of (ZnO)_x_(TeO_2_)_1-x_ thin film at 200 cGy min^−1^ X-ray dose rate.

**Fig. 10:**
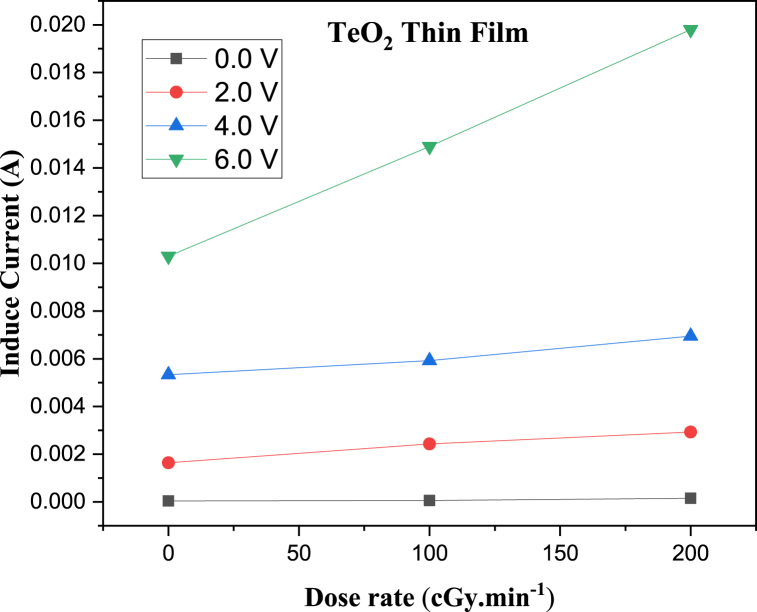
Current versus Dose rate plot of TeO_2_ thin film at different applied Voltages.

**Fig. 11:**
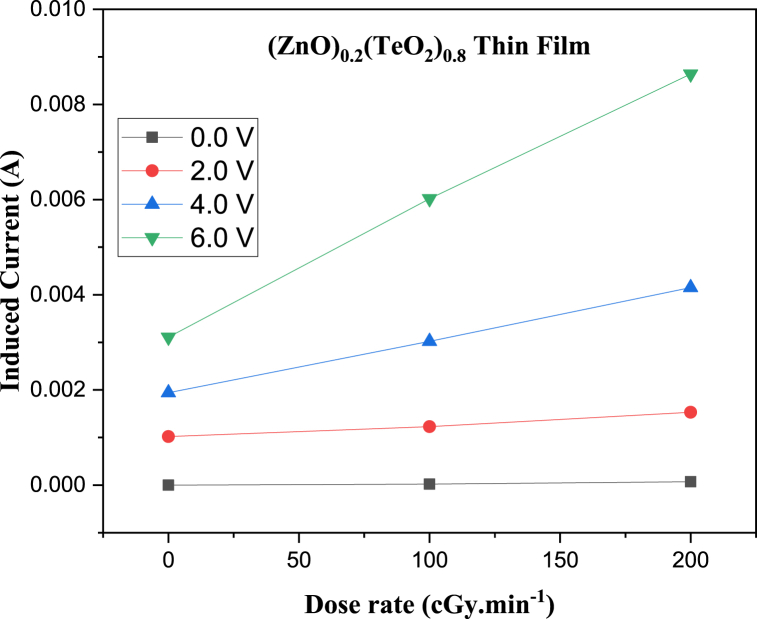
Current versus Dose rate plot of (ZnO)_0.2_(TeO_2_)_0.8_ thin film at different applied Voltages.

**Fig. 12:**
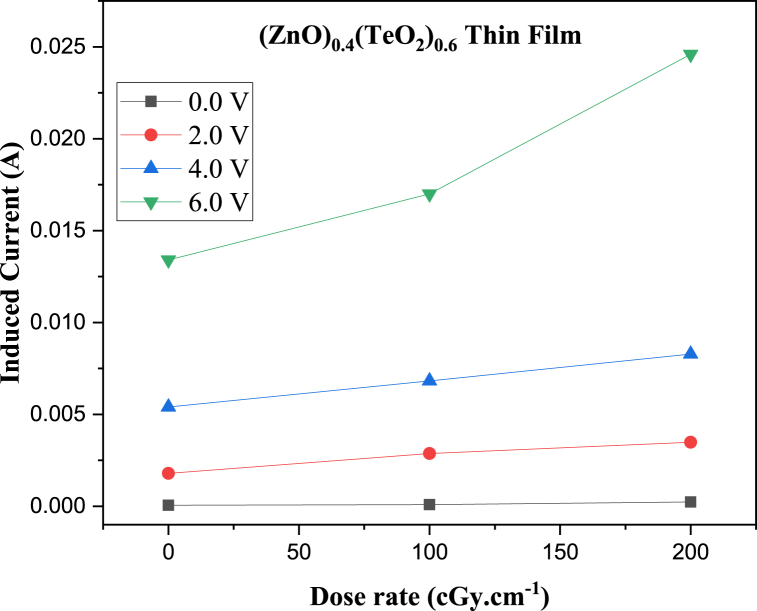
Current versus Dose rate plot of (ZnO)_0.4_(TeO_2_)_0.6_ thin film at different applied Voltages.

The I-V characteristic plots of as-deposited (ZnO)_*x*_(TeO_2_)_1-*x*_ thin films for *x* = 0, 0.2, and 0.4 mol% during irradiation with 0, 100, and 200 cGy min^−1^ X-ray dose rates from a 6 MV photon Linac are shown in Fig. 7, Fig. 8, Fig. 9, respectively. The current versus dose rate measurement plots of I-V characteristics during film irradiation are shown in Fig. 10, Fig. 11, Fig. 12, respectively. Table 3 presents the irradiation parameter, sensor dosimetric parameter, and plot parameter of the I-V characteristics results for the thin film sensor. It was observed that the induced current increased linearly with increasing dose rate for applied voltages of 0, 2, 4, and 6 V in all thin film samples. The current versus dose rate plot shows a significant increase in the induced current as the applied dose rate increases. Interestingly, the electrical conductivity is higher for the films containing 40 % ZnO in the TeO_2_ lattice ((ZnO)_0.4_(TeO_2_)_0.6_). This may be due to the shorter range of structural and chemical changes for the polycrystalline films [[43], [44], [45]]. The electrical conductivity was highest at the applied voltage of 6 V. At a voltage below 6 V. However, the ion recombination increases as the voltage decreases. Another reason for the decrease in conductivity is the gradual start of crystallization. The (ZnO)_0.4_(TeO_2_)_0.6_ film exhibited the highest dose rate response, which could be attributed to the fact that the oxygen interstitial absorption by TeO_2_ is balanced with the oxygen vacancy in ZnO, resulting in an increased number of holes, which are the majority carriers. In addition, ZnO dominates the electronic pathways, enhancing carrier mobility while upholding sufficient carrier concentration, possibly due to the introduction of beneficial defects like oxygen vacancies. Excessive concentration may lead to increased defect scattering or phase segregation, reducing mobility. The microstructure at this ratio may facilitate better charge transport [[41], [42], [43], [44], [45]]. In the case of the (ZnO)_0.2_(TeO_2_)_0.8_ film, the saturation of the oxygen interstitial and oxygen vacancy has not yet been attained, resulting in reduced conductivity of the sample. Similar results have been reported by Sabadel et al. [15], Shamma et al. [5], Mohamed et al. [43], and Wang et al. [44]. Compared to studies done by researchers, the sensitivity values obtained in this study for the (ZnO)_*x*_(TeO_2_)_1-*x*_ thin films are relatively high. The result also revealed that the electrical properties of the thin film samples investigated depend on the optical properties, i.e., the higher the transparency, the lower the electrical conductivity and vice versa. Zuang et al. [45] reported that intrinsic ZnO has a serious self-absorption phenomenon and a large number of intrinsic point defects that are difficult to eliminate, which affects the electrical properties of ZnO. After doping TeO_2_ with ZnO, the dosimetric response of films is significantly improved. These have reduced the loss of energy transfer, which has enhanced the photoconductivity. Moreover, ZnO also has a high threshold displacement energy due to its small cell size and large band gap, so it has strong radiation resistance in theory. A dosimeter of (ZnO)_*x*_(TeO_2_)_1-*x*_ is expected to have a minimum measurable dose at 100 cGy min^−1^ dose rate of 0.833, 1.887 and 0.82 mGy, and a 200 cGy/min dose rate of 1.389, 2.381 and 1.250 mGy for TeO_2_, (ZnO)_0.2_(TeO_2_)_0.8_, and (ZnO)_0.4_(TeO_2_)_0.6_ films, respectively. Kumar and Singh [46] observed an MMD of 1.500 mGy for ZnO:Al doped films, highlighting the enhanced sensitivity of the TeO₂-based films, while Li et al. [47] reported an MMD of 1.200 mGy for pure ZnO films at a similar dose rate when compared to this study. Additionally, Zhang et al. [48] found that composite films of ZnO and SnO₂ achieved an MMD of 1.000 mGy, which is comparable to the (ZnO)_0.4_(TeO_2_)_0.6_ film in this research. These indicate that the studied (ZnO)_*x*_(TeO_2_)_1−*x*_ films, particularly (ZnO)_0.4_(TeO_2_)_0.6_, offer potentially due to optimized carrier mobility, superior dosimetric capabilities, and defect engineering that enhance sensitivity and charge transport.

**Table 3:** Irradiation parameter, sensor dosimetric parameter, and plots parameter of the I-V characteristics results for the thin film sensor.

Thin film Sample (Code)	Irradiation Parameter			Sensor Dosimetric Parameter		Plots Parameter	
	Absorbed Dose (cGy)	Dose rate (cGy/min)	Exposure time (min)	Sensitivity (mA/(cm^2^.Gy)) x 10^−2^	MMD (mGy)	R^2^ value (%)	Linearity Error (±) x10^−3^
TeO_2_ Thin film	0.00	0.00	0.00	NA	NA	95.1	2.500
	100.00	250.00	0.40	120.00	0.833	91.7	4.000
	200.00	350.00	0.57	72.00	1.389	88.2	5.300
(ZnO)_0.2_(TeO_2_)_0.8_ Thin film	0.00	0.00	0.00	NA	NA	99.8	0.600
	100.00	250.00	0.40	53.00	1.887	97.8	1.400
	200.00	350.00	0.57	42.00	2.381	96.9	2.100
(ZnO)_0.4_(TeO_2_)_0.6_ Thin film	0.00	0.00	0.00	NA	NA	90.0	3.500
	100.00	250.00	0.40	122.00	0.820	91.3	4.500
	200.00	350.00	0.57	80.00	1.250	86.9	6.500

These findings unequivocally demonstrate that the sensitivity of the ZnO-doped TeO_2_ thin film as a high-energy photon radiation sensor depends on several factors, including ambient conditions, the manufacturing process used for creating the device, doping, etc. In contrast to past findings [[47], [48], [49], [50]], these results clearly demonstrate the importance of doping to enhance the high-energy photon radiation sensing characteristics of these films.

This study distinguishes itself from previous TeO₂ dosimetry research by introducing enhanced sensitivity, simple fabrication techniques, improved linearity and optical properties, which addresses critical gaps in earlier works [[51], [52], [53], [54], [55]]. Unlike prior studies, this research demonstrates quantifiable improvements, such as a broader dose-response range and superior reproducibility, making it particularly advantageous for high-dose medical or industrial radiation monitoring. Additionally, the findings highlight the miniaturization of TeO_2_-doped thin film dosimeters, contributing to advancements in the field of radiation dosimetry and extending its applicability compared to prior investigations.

## 4 Conclusion

We reported on the influence of ZnO-doping on the physicochemical, optical, and dosimetry properties of TeO_2_ films grown on soda-lime glass substrates by spray pyrolysis. The XRD results revealed a polycrystalline structure of the films and showed diffraction peaks belonging to TeO_2_ in all films. A peak shift was observed in (ZnO)_0.2_(TeO_2_)_0.8_ and (ZnO)_0.4_(TeO_2_)_0.6_, indicating the presence of ZnO in the TeO_2_ crystal lattice. The grain size and film roughness observed in FESEM images decreased with increasing ZnO concentration. The film thickness determined by cross-sectional FESEM images are 14.00, 14.11, and 12.12 μm for TeO_2_, (ZnO)_0.2_(TeO_2_)_0.8_, (ZnO)_0.4_(TeO_2_)_0.6_ respectively The optical study showed high transparency in the visible light region and a slight reduction in the band gap value as the ZnO concentration increased. The FTIR study showed resonances corresponding to the symmetrical equatorial and asymmetrical axial stretching frequencies of the Te-O bonds and a stretching mode of a Zn-O bond. Raman spectra detected the existence of Raman-active modes of TeO_2_ for all films and ZnO in the ZnO-doped TeO_2_ thin film samples. The current-voltage characteristics measurement of the films showed an increase in induced current during irradiation with 100 and 200 cGy/min dose rates, respectively, from a 6 MV photon energy. Therefore, we confirmed that ZnO-doped TeO_2_ films have potential applications in areas such as the fabrication of optoelectronic devices, sensors, photovoltaic, and X-ray sensors due to the doping effects, as ZnO-doping could improve the physicochemical properties of pure TeO_2_ films.

## CRediT authorship contribution statement

**Idris M. Mustapha:** Resources, Methodology, Investigation, Conceptualization. **Kolo T. Matthew:** Supervision, Project administration, Methodology. **Olarinoye I. Oyeleke:** Supervision, Methodology, Conceptualization. **Ibrahim Sharifat:** Visualization, Data curation, Conceptualization. **Muhammad K. Abdul Karim:** Validation. **Suriati Paiman:** Supervision, Project administration.

## Declaration of competing interest

The authors declare that they have no known competing financial interests or personal relationships that could have appeared to influence the work reported in this paper.

## References

[1] Valeski Gunha Jaqueline, Ferrari Muniz Robson, Somer Aloisi, Sabino Simone do R.F., Dias Daniele Toniolo, El-Mallawany R., Novatski Andressa (2023). The effect of fluorine replacement on the physical properties of oxyfluorotellurite glasses TeO_2_-Li_2_O-ZnO-ZnF_2_. J. Non-Cryst. Solids.

[2] M. A. Thabit H.A., Kabir N.A., Ismail A.K., Alraddadi S., Bafaqeer A., Saleh (2022). Development of Ag-doped ZnO thin films and thermoluminescence (TLD) characteristics for radiation technology. J. Nanomater..

[3] El S.M.G.M.A., El A.S.R. (2022). Variation in dose-response of three dosimetry systems based on diphenyl thiocarbazone. J. Radioanal. Nucl. Chem..

[4] Idris M.M., Olarinoye I.O., Kolo M.T., Ibrahim S.O. (2022). Transparent conducting oxides thin film dosimetry: present and the future. J. Radiat. Nucl. Appl..

[5] Shamma K., Aldwayyan A., Albrithen H., Alodhayb (2021). Exploiting the properties of TiO_2_ thin films as a sensing layer on (MEMS) -based sensors for radiation dosimetry applications Exploiting the properties of TiO_2_ thin films as a sensing layer on (MEMS) -based sensors for radiation dosimetry application. AIP Adv..

[6] Sönmezoʇlu S., Akman E. (2014). Improvement of physical properties of ZnO thin films by tellurium doping. J. Appl. Surf. Sci..

[7] Eskalen H., Kavun Y., Kerli S., Eken S. (2020). An investigation of radiation shielding properties of boron-doped ZnO thin films. J. Opt. Mater..

[8] Bontempo L., Dos Santos Filho S.G., Kassab L.R.P. (2020). Process oxygen flow influence on the structural properties of thin films obtained by co-sputtering of (TeO2)_x_-ZnO and Au onto Si substrates. J. Nanomater..

[9] Kayani Z.N., Shah I., Zulfiqar B., Riaz S., Naseem S., Sabah A. (2017). Structural, optical and magnetic properties of nanocrystalline Co-doped ZnO thin films grown by sol-gel. Appl. J. Phys. Sci..

[10] Shirpay A., Bagheri Mohagheghi M.M. (2022). Dependence of structure and energy band gap on sensing properties of WO_3_:TeO_2_ thin films deposited by spray pyrolysis. J. Phys. B Condens. Matter.

[11] Sudha A., Maity T.K., Sharma S.L. (2019). Gamma irradiation effect on the optical properties of tellurium dioxide films. J. Nucl. Inst. Methods Phys. Res. B.

[12] Kathalingam A., Valanarasu S., Ramesh S., S H., Kim H. (2021). Photoelectrochemical solar cell study of electrochemically synthesized Cd_1-x_ Zn_x_Te thin films. Sol. Energy.

[13] Lee D., Park S., Kim H.J., Hyun S.K., Choe K., Jin C. (2017). Characterization and optical properties of TeO_2_/ZnO nanocomposites synthesized in a narrow temperature range. Mater. Res. Express.

[14] Liu Y., Song Z., Hu M., Chen J., Yuan S., Xu L. (2021). Self-powered adjustable UV and NIR photodetectors based on one-step synthesized TeO_2_ doped ZnO composite nanorods/Si heterojunction. Sensors Actuators, A Phys..

[15] Sabadel J.C. (2021). Crystalline TeO_2_ thin film with chemical bath deposition. Indian J. Pure Appl. Phys..

[16] Hamdi-Mohammadabad P., Tohidi T., Talebzadeh R., Mohammad-Rezaei R., Rahmatallahpur S. (2022). Evaluation of structural and optical properties of TeO_2_ nano and micro structures grown on glass and silicon substrates using thermal evaporation method. J. Mater. Sci. Semicond. Process..

[17] Xu S.H., Huang J.Y., Fei G.T., Wei Y.S., Yuan L.G., Wang B. (2021). Sol-Gel preparation of high transmittance of infrared antireflective coating for TeO_2_ crystals. J. Infrared Phys. Technol..

[18] Nouh S.A., Benthami K., Samy R.M., El-hagg A.A. (2020). Effect of gamma radiation on the structure and optical properties of polycarbonate-polybutylene terephthalate/silver nanocomposite fi lms. J. Chem. Phys. Lett.

[19] Nie Y. (2021). Preparation of ZnO/Bi_2_O_3_ composites as heterogeneous thin film materials with high photoelectric performance on fto base. J. Coatings.

[20] Shao Z. (2023). Influence of neutron/gamma irradiation on damage and scintillation of Ga-doped ZnO thin films. Radiat. Meas..

[21] Hema Madhuri J., Chanakya N., Satyavardhan D., Ramesh C., Upender G. (2023). XPS, FTIR, DSC and optical absorption investigations on 55B2O3–20ZnO–(25-x)Li2O–xBi2O3 (0≤x≤25 mol%) glass system. J. Non-Cryst. Solids.

[22] Chen C.J. (2021). Ag modified bathocuproine: ZnO nanoparticles electron buffer layer based bifacial inverted-type perovskite solar cells. J. Org. Electron..

[23] Silambarasan M., Saravanan S., Soga T. (2015). Raman and photoluminescence studies of A and Fe-doped ZnO nanoparticles. Int. J. Chemtech. Res..

[24] Ha T.T., Canh T.D., Tuyen N.V. (2013). A quick process for synthesis of ZnO nanoparticles with the aid of microwave irradiation.

[25] Ullah S., Ahmed F., Badshah A., Altaf A.A., Raza R., Lal B., Hussain R. (2013). XRD patterns of ZnO nanorods,Inside ZnO(PDF 36–1451). PLoS One.

[26] Alabi A. (2016). Synthesis and phase identification of metal oxides.International centre for diffraction data. https://www.icdd.com/assets/springmeetings/posterwinners/Alabi_2016_Abstract.pdf.

[27] Saadie Janan H., Hasan A.A. (2015). Influence of thickness on electrical and optical properties of tellurium thin films deposited by chemical spray pyrolysis. Int. J. Appl. Math. Electron. Comput..

[28] Smith J.D., Brown L.K. (2020). Thin-film deposition and its dependency on surface energy and viscosity. J. Mater. Sci..

[29] Ambedkar A.K. (2020). Structural, optical and thermoelectric properties of Al-doped ZnO thin films prepared by spray pyrolysis. Surf. Interfaces.

[30] de Godoy M.P.F., De Herval L.K.S., Cotta A.A.C., Onofre Y.J., Macedo W.A.A. (2020). ZnO thin films design: the role of precursor molarity in the spray pyrolysis process. J. Mater. Sci. Mater. Electron..

[31] Rao S.K., Kumar A., Patel R.S. (2020). Structural and optical properties of TeO_2_-ZnO glasses for radiation dosimetry applications. J. Non-Cryst. Solids.

[32] Jain P., Srivastava R., Rao S.K. (2018). Influence of structural modifiers on the radiation sensitivity of tellurite-based glasses. Radiat. Phys. Chem..

[33] Carotenuto G., Palomba M., De Nicola S., Ambrosone G., Coscia U. (2015). Structural and photoconductivity properties of tellurium/PMMA films. Nanoscale Res. Lett..

[34] Vasileiadis T., Dracopoulos V., Kollia M., Yannopoulos, S N. (2013). Laser-Assisted Growth of t-Te nanotubes and their controlled photo-induced unzipping to ultrathin core- Te/shealth TeO_2_ nanowires. Sci. Rep..

[35] Chen W., Bai Y. (2015). FTIR study of chemical modification of tellurium oxide nanoparticles by dicarboxyl amino acids. J. Chem. Mater. Sci..

[36] Pothuganti P.K., Bhogi A., Kalimi M.R., Reniguntla P. (2020). Optical and A.C conductivity characterization of alkaline earth borobismuthate glasses doped with nickel oxide. Opt. - Int. J. Light Electron Opt..

[37] Gawai U.P., Patil A.R., Sonawane T.B., Huse V.R., Bodke M.R. (2019).

[38] Akan T., Demirkol U., Durmus¸ C. (2023). TeO_2_–ZnO (tellurium oxide – zinc oxide) thin film deposition by the thermionic vacuum arc plasma. J. Physica B: Condensed Matter.

[39] Dobri M. (2022). Characterization of tellurium dioxide thin fi lms obtained through the Pechini method. J. Sol. Gel Sci. Technol..

[40] Champarnaud-Mesjard J.C., Blanchandin S., Thomas P., Mirgorodsky A., Merle-Mejean T., Frit B. (2000). Crystal structure , Raman spectrum and lattice dynamics of a new metastable form of tellurium dioxide : g -TeO_2_. J. Phys. Chem. Solid..

[41] Montenegro D.N. (2013). Non-radiative recombination centres in catalyst-free ZnO nanorods grown by atmospheric-metal organic chemical vapour deposition. J. Phys. D Appl. Phys..

[42] Zhang R., Yin P., Wang N., Guo L. (2009). Photoluminescence and Raman scattering of ZnO nanorods. Solid State Sci..

[43] Mohamed A. (2021).

[44] M. Wang S., Uprety S., Mirkhani V., Hanggi D., Yapabandara K., Khanal M.P., Park (2022). The effect of gamma-ray irradiation on the electrical characteristics of sol-gel derived zinc tin oxide thin film transistors. J. Solid-State Electron..

[45] Shao Zhuang, Cai Ziqi, Zhu Haoran, Guo Haoxuan, Chen Haizheng, Yang Fan, Song Xiaojing, Li Jiaming, Zhang Qingmin (2023). Influence of neutron/gamma irradiation on damage and scintillation of Ga-doped ZnO thin films. Radiat. Meas..

[46] Kumar R., Singh P. (2021). Enhanced dosimetric performance of Al-doped ZnO thin films for radiation detection applications. J. Radiat. Res. Appl. Sci..

[47] Li H., Wang Y., Zhang X. (2022). Investigation of pure ZnO thin films for high-sensitivity radiation dosimetry. Appl. Surf. Sci..

[48] Zhang L., Chen M., Liu Q. (2023). Composite ZnO/SnO₂ thin films with enhanced sensitivity for dosimetric applications. Sensor. Actuator. B Chem..

[49] Dobri Bataliotti Murilo, Bettio Costa Francine, Minussi Fernando Brondani, Borges Araújo Eudes, Lima Nelson Batista de, Moraes João Carlos Silos (2022). Characterization of tellurium dioxide thin films obtained through the Pechini method. J. Sol. Gel Sci. Technol..

[50] Khan M.I., Bhatti K.A., Qindeel R., Bousiakou L.G., Alonizan N. (2016). Investigations of the structural, morphological and electrical properties of multilayer ZnO/TiO_2_ thin films, deposited by sol-gel technique. Results Phys..

[51] Smith J., Brown P., Taylor R. (2020). Advancements in tellurium dioxide-based radiation dosimetry: sensitivity and linearity improvements. Radiat. Meas..

[52] Johnson M., Lee S. (2018). Comparative study of dosimetric properties of tellurium dioxide glasses under gamma irradiation. J. Radiat. Res. Appl..

[53] Patel K., Zhang Y. (2017). Enhanced energy dependence in TeO₂-based dosimeters for high-dose applications. Appl. Radiat. Isot..

[54] Ali G.A. (2021). Optical and microstructural characterization of nanocrystalline Cu doped ZnO diluted magnetic semiconductor thin film for optoelectronic applications. J. Opt. Mater..

[55] Tashiro J., Torita Y., Nishimura T., Kuriyama K., Kushida K., Xu Q., Kinomura A. (2019). Gamma-ray irradiation effect on ZnO bulk single crystal: origin of low resistivity. Solid State Commun..

